# Short sleep duration in adults with congenital heart disease is associated with epicardial adipose tissue accumulation

**DOI:** 10.3389/fpsyt.2025.1490564

**Published:** 2025-09-17

**Authors:** Britta Stapel, Lotta Winter, Ivo Heitland, Tim Halling, Steffen Akkermann, Jochen Muke, Friederike Löffler, Johann Bauersachs, Mechthild Westhoff-Bleck, Kai G. Kahl

**Affiliations:** ^1^ Department of Psychiatry, Social Psychiatry and Psychotherapy, Hannover Medical School, Hannover, Germany; ^2^ Department of Cardiology, Fachklinik Bad Bentheim, Bad Bentheim, Germany; ^3^ Department of Cardiology and Angiology, Hannover Medical School, Hannover, Germany

**Keywords:** sleep duration, epicardial adipose tissue, NT-ProBNP, adult congenital heart disease, ACHD

## Abstract

Short sleep duration has been linked to an increased risk of cardiometabolic disorders and adverse outcomes, including hospitalization and mortality, in patients with cardiovascular disease (CVD). We assessed the association between sleep duration and cardiovascular parameters in adults with congenital heart disease (ACHD). Data were derived from the ongoing PSYConHEART study on morbidity and mortality factors in ACHD. Sleep duration, sociodemographic variables, and symptoms of anxiety and depression were measured using self-report questionnaires. Epicardial adipose tissue (EAT) was measured by echocardiography, and clinical parameters regarding the underlying heart condition, including serum levels of N-terminal pro-B-type natriuretic peptide (NT-proBNP), were assessed. Patients with a score < 3 on the Hospital Anxiety and Depression Scale depression subscale were included (*N* = 194). Short sleep duration (≤ 6 h/night) was present in 48 patients (25%). Two-way multivariate analysis with sleep duration (≤ 6 h/night vs. > 6 h/night) and age (≥ 35 vs. < 35 years) as independent variables, corrected for BMI, sex, and NYHA class, revealed a significant effect of sleep duration and age on prognostic CVD markers, namely EAT and NT-proBNP. Sleep duration was associated with CVD markers in older patients only. Sleep duration was associated with CVD markers only in older patients. In particular, EAT, which has prognostic value in cardiac diseases, was negatively impacted by short sleep duration. Sleep problems/disorders are amenable to psychological and pharmacological interventions. Therefore, assessment of sleep problems/disorders may be recommended as part of the multimodal treatment of ACHD patients.

## 1 Introduction

Congenital heart disease (CHD) constitutes the most common congenital defect, affecting approximately 1% of all live births (1). Over the last decades, substantial improvements in early diagnostic, interventional, and surgical procedures have significantly increased survival rates of children with CHD, with over 95% now expected to reach adulthood (2). Consequently, adults with congenital heart disease (ACHD) constitute an ever-growing patient population.

While long-term prognosis for patients with ACHD has significantly improved, previous studies have reported higher rates of anxiety and depression in ACHD patients compared to the general population (3, 4), and an increased risk of cardiovascular disease (CVD) has been identified in this population (5).

Given the heightened risk in ACHD patients for both psychological disorders and adverse cardiovascular outcomes, it is of major importance to identify additional, modifiable risk factors that might adversely impact their physical and mental well-being.

In this regard, the adverse effects of insufficient sleep quantity on both physical and mental health have been established (6, 7). Complaints of inadequate sleep have increased over recent decades, a trend attributed to adverse lifestyle habits that have become more prevalent in modern society, including extreme work schedules, lack of exercise, unhealthy diets, smoking, high levels of psychological stress, and an increased use of electronic devices (8). Short sleep duration, in particular, appears to be common, with nearly two-thirds of the adult population in the USA reporting sleep durations of 7 h or less (9). By contrast, the National Sleep Foundation in the USA defines adequate sleep for adults aged 18 to 65 years as 7 to 9 h per night (10).

Importantly, short sleep duration that persists for a considerable period, which might result from difficulties initiating sleep, maintaining sleep, or early awakenings, constitutes a core symptom of chronic insomnia (11, 12). Insomnia is often considered a transdiagnostic symptom across many mental disorders, including mood, psychotic, and anxiety disorders. In this regard, patients with mental illnesses report sleep disturbances with a frequency of 70%–80% of cases during the acute phase of their disorder (13). However, insomnia has been shown to be an independent risk factor for the development of mental disorders and also has been established as an independent risk factor for physical illnesses, including CVD (14, 15). Consequently, insomnia is considered a mental disorder according to the current classification in the *International Statistical Classification of Diseases and Related Health Problems (ICD)-10* (16) and has been included in the *Diagnostic and Statistical Manual of Mental Disorders (DSM)* since 1987 (17). Accordingly, cognitive behavioral therapy for insomnia (CBT-i) is recommended as the first-line treatment for patients diagnosed with insomnia (11).

Furthermore, sleep disorders are highly prevalent in the general population (18) and appear even more frequent in patient populations with mental or physical illnesses (19, 20). In patients with established CVD, studies suggest a bidirectional relationship between sleep disorders and CVD progression. On one hand, sleep disorders, including chronic insomnia, are more frequent in patients with CVD than in the general population (19, 21) and have been associated with adverse outcomes in these patients (22). On the other hand, sleep disorders and inadequate sleep quality or duration have been associated with an increased risk of cardiometabolic diseases that predispose to CVD, as well as with a higher risk of CVD itself (23).

Against this background, sleep parameters and their association with prognostic CVD markers remain understudied in patients with ACHD. Previous research has focused primarily on pathological alterations in sleep quantity or quality due to underlying somatic causes, reporting an increased prevalence and severity of obstructive sleep apnea (OSA), which has been associated with short sleep duration in patients with ACHD (24, 25).

Body fat distribution is a predictive marker for CVD risk. In particular, epicardial adipose tissue (EAT) has been extensively studied in the context of CVD because of its close proximity to the coronary arteries and the myocardium, suggesting potential paracrine or vasocrine crosstalk (26). EAT has been established as an early predictor of cardiovascular (CV) morbidity and mortality (26). Increased cardiac adipose tissue has been associated with common risk factors, as well as with a higher risk of type 2 diabetes, atherosclerotic CVD, and heart failure; it has also been negatively associated with CV health scores (26–28).

In the context of ACHD, previous studies have reported increased EAT content in young patients with major depressive disorder (29). Additionally, a recent study established a link between adverse childhood experiences and EAT, mediated by depressive symptoms and reduced physical activity in patients with ACHD (30).

Alongside EAT, the neurohormone N-terminal pro-B-type natriuretic peptide (NT-proBNP) is one of the best-studied biomarkers in patients with CVD, including those with ACHD. NT-proBNP is a stable amino acid fragment released into the circulation by ventricular cardiomyocytes in response to stretch (31). Serum NT-proBNP levels have been shown to be strongly associated with CV events and mortality risk, particularly in the context of heart failure (32). Furthermore, in a nationally representative sample of adults without a history of CVD, NT-proBNP levels in the top quartile were associated with a significantly increased risk of both CV and all-cause mortality (33). In clinically stable patients with ACHD, NT-proBNP was strongly associated with CV events, independent of electrographic and echocardiographic measurements as well as clinical characteristics (34). Moreover, serial NT-proBNP testing has been reported to help identify ACHD patients at risk for adverse CV events (35).

Finally, research investigating the potential association between sleep disorders and EAT content has been largely limited to patients with OSA. These studies commonly report increased EAT thickness in OSA patients and an association of EAT with OSA severity (36). In contrast, prior studies examining the impact of OSA, and particularly of OSA treatment, found no significant impact on NT-proBNP levels (37). Furthermore, literature regarding the association between insomnia or insomnia symptoms and NT-proBNP levels appears limited and yielded heterogeneous results (38, 39).

We are not aware of any previous works that have assessed EAT thickness or NT-proBNP serum levels in relation to sleep duration in patients with ACHD.

We hypothesize that short sleep duration, defined as 6 h or less of total sleep per night, adversely impacts CV health in patients with ACHD. To test this hypothesis, we assessed the association of self-reported short sleep duration with prognostic markers of heart failure (i.e., EAT thickness and serum levels of NTproBNP) in ACHD patients without present depressive symptoms, as indicated by a score of less than 3 on the depression subscale of the Hospital Anxiety and Depression Scale (HADS-D < 3).

## 2 Methods

### 2.1 Study design and participants

Data included in this work were derived from the ongoing PSYConHEART study, which focuses on identifying factors associated with morbidity and mortality in a sample of ACHD patients. Findings from the PSYConHEART study have been reported in several prior publications (3, 30, 40–42). However, the association between sleep duration and prognostic markers of CVD has not previously been assessed. The study was approved by the local ethics committee at Hannover Medical School and conducted in accordance with the Declaration of Helsinki. Written informed consent was obtained from all study participants. Patient recruitment was conducted at the ACHD outpatient clinic of the Department of Cardiology and Angiology at Hannover Medical School. General inclusion criteria for the PSYConHEART study were as follows: (a) diagnosis of a structural congenital heart disease, (b) ability to read and understand the self-report questionnaires, and (c) age 18 years or older. Exclusion criteria included pregnancy and/or instability of the underlying heart condition.

Additional exclusion criteria were applied to the sample included in the present study, based on the research question focusing on the association between short sleep duration and CVD markers. According to the literature, short sleep duration was defined as 6 h per night or less over the previous month, as indicated by a self-report questionnaire (10). As respective recommendations for sleep duration are directed toward adults aged 18 to 65 years, participants older than 65 years were excluded from the analyses. In addition, patients who displayed significant depressive symptoms at study entry, defined as an HADS-D score ≥ 3, were excluded to prevent potential confounding from depression, given previously reported interactions between depression and sleep disorders (6). Indeed, before excluding patients with depressive symptoms, HADS-D scores differed significantly between groups defined by sleep duration (sample size: *N* = 424; *U* = 16,111.0, *Z =* − 2.330, *p* = 0.020). The corresponding mean values and standard deviations are shown in **Supplementary Table S1**. The HADS-D cut-off value was defined according to the literature, which determined a HADS-D score > 2 as indicative of mild depression in ACHD patients (43). **Supplementary Figure S1** provides detailed information on the sample selection.

### 2.2 Cardiovascular evaluation and echocardiographic assessment of EAT

Examination of all patients was conducted by a senior cardiologist certified as an expert in ACHD during routine check-ups. The complexity of the underlying congenital heart defect was classified using the Bethesda scale as “simple”, “moderate”, or “complex” (44). Functional symptoms of heart failure were assessed in accordance with the New York Heart Association (NYHA) Functional Classification, with NYHA I indicating no limitation, NYHA II slight limitation, and NYHA III marked limitation of physical activity, and NYHA IV indicating an inability to carry out any physical activity without discomfort. Anthropometric measures, including weight and height, along with standard laboratory parameters such as N-terminal pro-B-type natriuretic peptide (NT-proBNP), were assessed.

Thickness of EAT was assessed using echocardiography as follows: two-dimensional standard parasternal long- and short-axis images were obtained at end-diastole. Measurements were conducted on the right ventricular free wall, perpendicular to the aortic annulus (29).

### 2.3 Self-report questionnaires

Effective sleep duration per night was assessed using a self-report questionnaire item asking about sleep over the previous 4 weeks. Symptoms of depression and anxiety were measured with the depression and anxiety subscales of the German version of the HADS, and total HADS sum scores were also calculated (45). The HADS is a self-report questionnaire comprising seven items for each of the depression and anxiety subscales. Regarding the depression subscale, five of the seven items relate to the loss of pleasure response. Each item is scored on a 4-point scale from 0 to 3, resulting in a maximum score of 21 for each subscale. Internal consistency (Cronbach’s α) for the English and German versions of the HADS has been reported to be acceptable, ranging from 0.80 to 0.93 for the anxiety subscale and from 0.81 to 0.90 for the depression subscale (46). Cut-off values in this study were derived from a dedicated publication that defined modified cut-off values for patients with ACHD (43).

### 2.4 Statistical analyses

Statistical analyses were performed using SPSS 28 (IBM, Armonk, NY, USA). To test for normal distribution of data, the Shapiro–Wilk test was performed. For group comparisons of anthropometric and demographic data, as well as psychological variables, the nonparametric Mann–Whitney *U* test was applied, while the Chi-square test was used for comparisons of nominal data. Age has previously been reported as a factor associated with EAT thickness (47, 48), serum levels of NT-proBNP (49), and sleep duration (10, 50). In the present sample, age differed significantly between patient groups defined by sleep duration (**Table 1**). Additionally, a significant interaction effect was found between sleep duration (dependent variable) and age (covariate) when testing assumptions for the multivariate analysis of covariance (MANCOVA) described below. Therefore, age could not be included as a potential confounder in the model. Age groups were subsequently derived by dichotomization of the variable age using a median split. To test a potential association of sleep duration and age group with EAT thickness and NT-proBNP levels, a two-tailed MANCOVA was performed with EAT and NT-proBNP as combined dependent variables, and sleep duration and age group as independent variables, controlling for BMI, NYHA class, and sex. Groupwise comparisons were conducted using Bonferroni-corrected *post hoc* tests. Two-tailed *p*-values are reported, and *p* ≤ 0.050 was considered statistically significant.

**Table 1:** Group comparison of sociodemographic characteristics and CV measures in ACHD patients stratified by sleep duration.

	Sleep duration		Statistics	
	> 6 h (*N* = 146)	≤ 6 h (*N* = 48)	Effect size	*p*-value
Age (years)	32.5 [26–41]	39.5 [31–50]	*U* = 2,453.5, *Z* = − 3.115	*p*=.002
Female (*N* [%])	74 (51%)	19 (40%)	*χ*² (1) = 1.784, *φ* = − 0.096	*p*=182
BMI (kg/m^2^)	24.0 [21.7–26.9]	25.0 [22.0–27.0]	*U* = 3,188.5, *Z* = − 0.935	*p*=.350
Bethesda scale			*χ*² (2) = 0.515, *φ* = 0.052	*p*=.773
Bethesda I (*N* [%])	12 (8%)	3 (6%)		
Bethesda II (*N* [%])	58 (40%)	17 (36%)		
Bethesda III (*N* [%])	75 (52%)	27 (57%)		
NYHA classification			*χ*² (2) = 4.548, *φ* = 0.153	*p*=.103
NYHA I (*N* [%])	116 (80%)	31 (65%)		
NYHA II (*N* [%])	26 (18%)	14 (29%)		
NYHA III (*N* [%])	4 (3%)	3 (6%)		
Sleep duration (h/night)	7.5 [7.0–8.0]	6.0 [5.0–6.0]	*U* = 0.000, *Z* = − 10.521	*p*<.001
HADS depression scale	1 [1.0–2.0]	1 [0.25–2.0]	*U* = 3.426, *Z* = − 0.249	*p*=.803
HADS anxiety scale	4 [2.0–6.0]	4 [2.0–6.0]	*U* = 3,176.5, *Z* = − 0.636	*p*=.525
HADS sumscore	5 [3.0–7.0]	5 [3.0–7.0]	*U* = 3,182.5, *Z* = − 0.617	*p*=.537

Unless otherwise indicated, median and interquartile range (IQR) are shown, and asymptotic two-tailed *p*-values are reported. *p* ≤ 0.05 was considered statistically significant. *BMI*, body mass index; *HADS*, hospital anxiety and depression scale; *NYHA class*, New York Heart Association Functional classification.

## 3 Results

### 3.1 Patient characteristics of the complete sample

A total of 194 patients with ACHD were included in the final study sample. The median age of the sample was 35 years (interquartile range [IQR]: 27–42; min–max: 18–65), and 93 (48%) of the patients were women. Most patients had no or mild functional symptoms of heart failure (NYHA class I: *N* = 147 [76%]; NYHA class II: *N* = 40 [21%]), while fewer patients presented with moderate symptoms (NYHA class III: *N* = 7 [4%]); no patients with severe symptoms were present. According to the Bethesda classification, as a measure of the complexity of the underlying congenital heart defect, most patients presented with complex defects (*N* = 102 [53%]), while 75 (39%) had moderate defects, and 15 had simple defects (8%). Patients reported a median sleep duration of 7 h per night (IQR: 6.4–7.8; min–max: 4–10), and 48 (25%) patients reported a sleep duration of 6 h or less per night for most nights of the previous month. Frequencies of CV medications in the study sample are provided in the **Supplementary Table S2**.

### 3.2 Comparison of sociodemographic variables and CV measures in ACHD patients based on sleep duration

In accordance with dedicated literature, patients were grouped based on self-reported sleep duration into those with sufficient sleep duration of more than 6 h per night and those with short sleep duration of 6 h or less per night. **Table 1** summarizes group comparisons of sociodemographic-, CV-, and psychological parameters. Groups did not differ significantly with regard to sex distribution, body mass index (BMI), severity of heart failure symptomology indicated by NYHA class, or complexity of the underlying heart condition in accordance with Bethesda class. Additionally, and as expected based on the defined HADS-D cut-off score, no significant differences were found regarding depressive symptomology. Furthermore, no significant differences were found in the anxiety subscale of the HADS and the HADS sum score. Patients who reported short sleep durations were significantly older (*U* = 2,453.5, *Z* = − 3.115; *p* = 0.002). Finally, while frequencies of prescription did not differ significantly for most CV medications depending on sleep duration (**Supplementary Table S3**), beta-blockers and pulmonary hypertension (PH) therapy were more frequently prescribed in patients with short sleep durations (beta-blockers: *χ*² (1) = 3.965, *p* = 0.046, *φ* = 0.143; PH therapy: *χ*² (1) = 5.844, *p* = 0.016, *φ* = 0.174; **Supplementary Table S3**).

### 3.3 EAT thickness and NT-proBNP levels in ACHD patients based on sleep duration and age

While literature data regarding an association of EAT with age are heterogeneous, some studies suggest increased age-dependent EAT accumulation, particularly in younger adults (47, 48). Furthermore, age-dependent effects on circulating levels of NT-proBNP have been described (49). Therefore, age should be considered in the analyses. However, the significant group differences regarding age prevented its inclusion as a covariate in the MANCOVA, as an interaction between age and sleep duration could not be excluded. Therefore, the variable age was dichotomized by median split, and subsequently, a two-way multivariate ANCOVA with EAT and NT-proBNP as dependent variables, sleep duration and age group as independent variables, and sex, BMI, and NYHA class as covariates was performed to assess a potential association of sleep duration with prognostic markers of CVD. The respective analysis showed a significant effect of sleep duration on the combined dependent variables (*F*[2, 186] = 4.290, *p* = 0.015, Wilk’s Λ = 0.956). Similarly, a significant difference between age groups on the combined variables was found (*F*[2, 186] = 6.129, *p* = 0.003, Wilk’s Λ = 0.938), and a significant interaction effect of sleep duration and age group was present (*F*[2, 186] = 4.888, *p* = 0.009, Wilk’s Λ = 0.950).

For both dependent variables, *post hoc* univariate ANCOVAs were conducted. A significant effect of sleep duration on EAT (*F*[1, 187] = 3.980, *p* = 0.047, *η*² = 0.021) as well as on NT-proBNP (*F*[1, 187] = 6.374, *p* = 0.012, *η*² = 0.033) was found. Furthermore, results indicate a significant difference between age groups for EAT (*F*[1, 187] = 9.552, *p* = 0.002, *η*² = 0.049) as well as for NT-proBNP (*F*[1, 187] = 5.169, *p* = 0.024, *η*² = 0.027). Finally, a significant interaction effect of sleep duration and age group was shown for NT-proBNP (*F*[1, 187] = 8.818, *p* = 0.003, *η*² = 0.045), but not for EAT (*F*[1, 187] = 2.571, *p* = 0.111, *η*² < 0.014).

**Figure 1:**
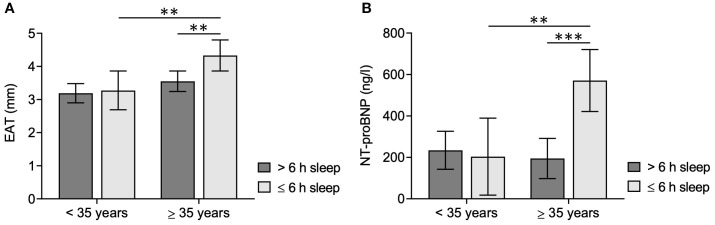
EAT and NT-proBNP levels in ACHD patients according to sleep duration and age group. Bar graphs show estimated marginal means and 95% confidence intervals (adjusted for BMI = 24.876, NYHA class = 1.28, and sex = 0.48) for **(A)** EAT and **(B)** NT-proBNP levels, stratified by sleep duration/night (> 6 h vs. ≤ 6 h) and age group (< 35 years vs. ≥ 35 years). Bonferroni-corrected two-tailed *p*-values indicate statistically significant pairwise group comparisons. Statistical significance was defined as *p* ≤ 0.05; ^***^ *p* ≤ 0.001; ^**^ *p* ≤ 0.010.

Pairwise group comparisons based on estimated marginal means were conducted using a Bonferroni-corrected *post hoc* test. A significant difference for EAT based on sleep duration was found only in the older age group (*p* = 0.006, *M*_Diff_ = 0.078; 95% confidence interval [CI] = 0.023, 0.134) but not in younger patients (*p* = 0.798, *M*_Diff_ = 0.008; 95% CI = − 0.057, 0.074). Similarly, sleep duration had a significant effect on NT-proBNP only in the older age group (*p* < 0.001, *M*_Diff_ = 376.4; 95% CI = 201.2, 551.6), while no significant effect was detected in the younger patient group (*p* = 0.769, *M*_Diff_ = − 30.7; 95% CI = − 236.5, 175.1). Results are visualized in **Figures 1A, B**.

To test the validity of these results, the analyses were repeated utilizing an expanded sample derived from a modified HADS-D cut-off value. By raising the cut-off from < 3 to < 6, thereby including patients reporting no or mild depressive symptoms, the sample size was increased to 337. The cut-off value was chosen based on dedicated literature data that defined a modified cut-off value of < 6 to identify ACHD patients with moderate to severe symptoms (43). The corresponding analyses confirmed the results presented above (see **Supplementary Results**; **Supplementary Tables S4**, **S5**).

## 4 Discussion

In the present work, we describe the association of short sleep duration with an adverse CV marker profile, indicated by increased EAT thickness and elevated levels of NT-proBNP, in individuals aged 35 years or older with ACHD.

Short sleep duration is defined as 6 h or less of effective sleep time per night for adults between the ages of 18 and 65 years (10). Together with prolonged sleep latency and daytime impairment, it constitutes one of the key symptoms of chronic insomnia (51). Short sleep duration is frequently reported in surveys of the general population. In this regard, the frequency of 25% of patients with ACHD who reported a sleep duration of 6 h or less in our sample was comparable to data from the general US population, which indicates frequencies of 28% (52). In the present sample, short sleep duration was associated with a higher frequency of beta-blocker and pulmonary hypertension treatment. In this regard, a recent meta-analysis concluded a possible increased risk for sleep disorders and sleep disturbances in the context of beta-blocker treatment (53). This is in line with older studies that found that beta-blockers decrease melatonin production via specific inhibition of the beta1-adrenoreceptor (54). Regarding pulmonary hypertension, our findings are consistent with the previously observed high frequency of poor sleep quality in affected patients (55).

Sleep duration is of importance in the context of cardiometabolic health, as inadequate sleep quantity, similarly to insomnia, has been attributed to adverse health behaviors, including increased calorie intake, and has been associated with the risk for cardiometabolic conditions, including obesity, diabetes mellitus, and hypertension (56, 57).

To our knowledge, measures of sleep duration or sleep quality and their associations with prognostic markers of CVD have not been investigated in patients with ACHD to date.

NT-proBNP contains the 76-amino-acid N-terminal fragment of BNP that is increasingly released from the heart in response to heightened myocardial wall stress (58). While NT-proBNP was originally considered a biomarker exclusively for heart failure (58), a prognostic value has been demonstrated in the context of ACHD. In this regard, NT-proBNP levels in the upper quartile were found to be associated with CV events and with heart failure or death in a sample of clinically stable patients with ACHD (median age of 33 years) (34). NT-proBNP levels have been previously studied in the context of sleep duration. However, data from the general population yielded ambiguous results. In this regard, one study suggested a significant association of subjective short sleep duration and NT-proBNP in elderly individuals above the age of 65 years (mean age = 76 years) and without a history of CVD (38). Contrarily, a second study that included a comparable age group found a significant effect of daytime sleep duration on NT-proBNP levels but failed to detect an effect of nighttime sleep (39). Overall, our finding that short sleep duration was associated with heightened levels of NT-proBNP, particularly in older patients with ACHD, is in line with the well-described adverse impact of short sleep duration and sleep deprivation on cardiometabolic health in the general population and on morbidity and mortality in patients with established CVD (22).

Similar to NT-proBNP, EAT constitutes a risk marker for cardiometabolic disorders (47, 59). Additionally, increased cardiac fat accumulation has been reported in the context of psychiatric disorders, including major depressive disorder (60). As psychiatric disorders are also linked to sleep disturbances, most noticeably insomnia, patients with relevant depressive symptoms (i.e., HADS-D ≥ 3) were excluded from the present study. Therefore, depression scores in this sample did not significantly differ in patients who reported short sleep duration compared to those who slept longer. Additionally, both patient groups were comparable regarding anxiety scores and retrospectively reported adverse childhood experiences (data not shown). This is important, as EAT thickness in patients with ACHD has previously been shown to be associated with depression, and a link between adverse childhood experiences, depression, physical activity, and EAT has been described (29, 30). Assessment of EAT and modifiable factors of EAT accumulation might be of high relevance, particularly in patients with ACHD, considering that the leading causes of death in this patient population include heart failure, ischemic heart disease, and arrhythmias (5, 61), which have previously been linked to cardiac adipose tissue accumulation (26, 62, 63).

Intriguingly, research that addressed EAT content in the context of sleep disturbances—including sleep duration, other insomnia symptoms, and insomnia itself—appears to be lacking with regard to the healthy population, CVD patients in general, and ACHD patients in particular. As EAT accumulation has previously been demonstrated to be affected by age, we dichotomized age in our sample by median split (47). While we found a significant effect of age group on EAT after controlling for confounders, this effect could be attributed to patients with short sleep duration. Contrarily, no significant difference between age groups was detected in patients with a sleep duration of more than 6 h per night. Similarly, sleep duration affected EAT thickness only in the older age group, indicating a significant effect of sleep duration on EAT in older patients.

## 5 Conclusion

Depending on the underlying congenital heart defect, patients with ACHD have at least the same—or an even higher—risk of developing CVD later in life compared with the general population. Residual issues, including valvular disease, shunts, ventricular dysfunction, and arrhythmias, contribute to an increased risk of heart failure. Therefore, identifying modifiable risk factors associated with CV risk indices in this patient population is crucial. Mounting evidence supports a significant association between sleep and CVD, leading to the proposal that sleep disturbances might be considered the 10th modifiable CV risk factor (64). Against this background, our findings that short sleep duration—one of the key symptoms of insomnia—was significantly associated with EAT thickness and NT-proBNP levels in older patients with ACHD are highly relevant. Although this study is preliminary and did not assess potential underlying causes of short sleep duration or other key symptoms of insomnia (i.e., daytime impairment or use of sleep medication), it suggests that assessment of sleep quality and quantity should be improved and incorporated into the multimodal management of ACHD patients.

## 6 Limitations

The present study has some limitations that should be considered. Due to the cross-sectional design, the study does not allow for temporal or causal inferences. Sleep duration was assessed using a single item in a self-report questionnaire, providing only subjective measures of sleep time. Additionally, we did not screen for potential underlying somatic conditions associated with short sleep duration, such as OSA, nor did we assess other indicators of poor sleep quality, including daytime functioning. Therefore, follow-up studies are planned to investigate the potential underlying causes of short sleep duration and the prevalence of sleep disorders in ACHD, as well as their respective impacts on cardiometabolic risk indices and CVD parameters.

In addition, the present study did not distinguish between patients with physiological sleep duration and those with long sleep duration within the group reporting at least 6 h of sleep per night. This distinction is relevant, as long sleep duration has been associated with an increased risk of CVD (65). However, in the present sample, only seven out of 194 patients reported long sleep duration, typically defined as ≥ 9 h/night. Due to this small sample size, the impact of long sleep duration was not assessed in the current study.

Finally, an additional important limitation regarding the statistical analysis concerns the handling of age. Due to violations of model assumptions, we applied a median split rather than including age as a continuous covariate. However, this approach is arbitrary, reduces statistical power, and may obscure continuous relationships. Future studies with larger samples should examine age as a continuous predictor or moderator.

## Data Availability

The raw data supporting the conclusions of this article will be made available by the authors, without undue reservation.
