# Supplementary material for: Short sleep duration in adults with congenital heart disease is associated with epicardial adipose tissue accumulation
Source: Front Psychiatry. 2025 Sep 17;16:1490564. doi: 10.3389/fpsyt.2025.1490564 (PMC12484156; doi:10.3389/fpsyt.2025.1490564)
Supplement: Supplementary file 1 [file DataSheet1.pdf]

Supplementary Material

1 Supplementary Figures

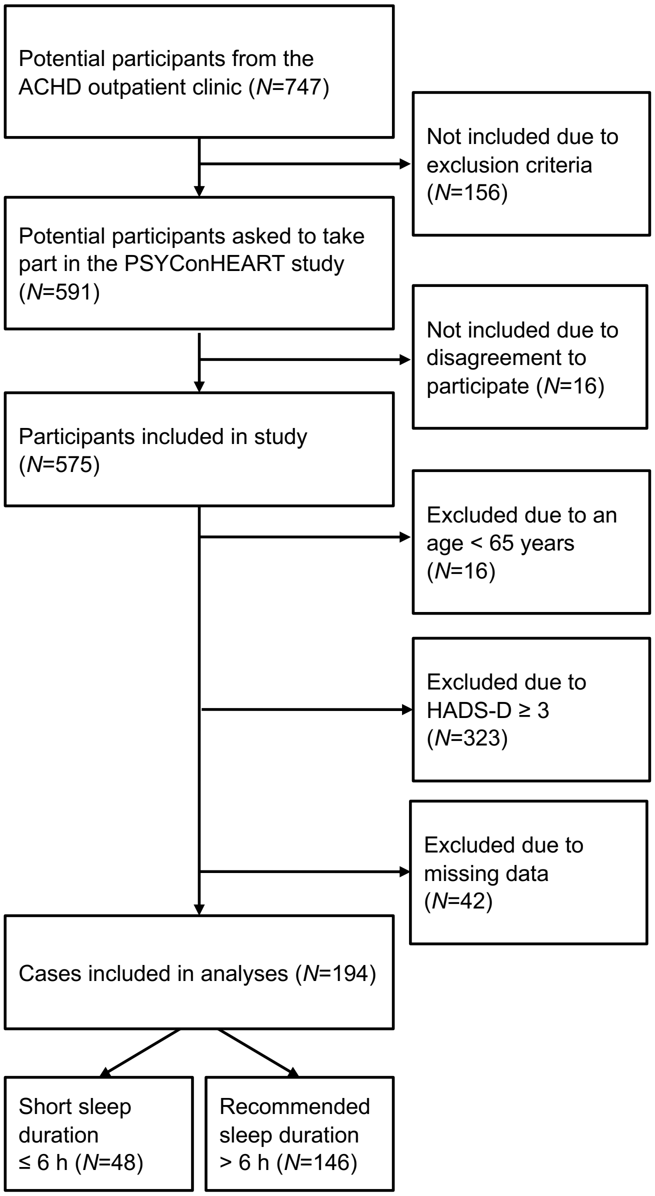

Supplementary Figure 1. Scheme detailing recruitment of patients and reasons for exclusion from the analyses.

2 Supplementary Tables and Results

Supplementary Table 1: HADS-D scores in ACHD patients before exclusion of patients reporting depressive symptoms

	**Sleep duration**			
	**> 6 h (*N*=298)**	**≤ 6 h (*N*=126)**	**Statistics**	***p*-value**
HADS Depression scale	3.37 ± 2.93	4.31 ± 3.66	*U*=16111.0, *Z*=-2.330	*p*=.020

Means ± standard deviations are depicted. Mann-Whitney U Test was performed and an asymptotic two-tailed *p*-value is shown. $p \leq .05$ was considered statistically significant.

Supplementary Table 2: Frequencies of cardiovascular medications in the study sample

**CV medication**	***N*=194**
Beta blockers [*N* (%)]	66 (34%)
ACE inhibitors and AT receptor blockers [*N* (%)]	62 (32%)
Anticoagulants [*N* (%)]	43 (22%)
Acetylsalicyclic acid [*N* (%)]	27 (14%)
Spironolactone/Eplerenone [*N* (%)]	27 (14%)
Diuretics [*N* (%)]	20 (10%)
L-thyroxine [*N* (%)]	14 (7.2%)
Lipid-lowering drugs [*N* (%)]	13 (6.7%)
Pulmonary hypertension therapy [*N* (%)]	6 (3.1%)
Specific antiarrhythmics [*N* (%)]	6 (3.1%)
Digoxin [*N* (%)]	5 (2.6%)
Calcium antagonists [*N* (%)]	4 (2.1%)
Entresto (*N* [%])	4 (2.1%)
Oral corticosteroids [*N* (%)]	4 (2.1%)
Antidiabetics [*N* (%)]	3 (1.5%)
Other antihypertensive medication [*N* (%)]	3 (1.5%)
Insulin [*N* (%)]	2 (1.0%)
Immunosuppressants [*N* (%)]	1 (0.5%)

ACE, angiotensin converting enzyme; AT, angiotensin; CV, cardiovascular.

Supplementary Table 3: Group comparison of CV medication in ACHD patients based on sleep duration

	Sleep duration			
**CV medication**	**> 6 h (*N*=146)**	**≤ 6 h (*N*=48)**	**Statistics**	***p*-value**
Beta blockers [*N* (%)]	44 (30%)	22 (46%)	$\chi^2(1)=3.965$, φ=.143	*p*=.046
ACE inhibitors and AT receptor blockers [*N* (%)]	45 (31%)	17 (35%)	$\chi^2(1)=.351$, φ=.043	*p*=.554
Anticoagulants [*N* (%)]	32 (22%)	11 (23%)	$\chi^2(1)=.021$, φ=.010	*p*=.885
Acetylsalicyclic acid [*N* (%)]	20 (14%)	7 (15%)	$\chi^2(1)=.024$, φ=.011	*p*=.878
Spironolactone/Eplerenone [*N* (%)]	17 (12%)	10 (21%)	$\chi^2(1)=2.546$, φ=.115	*p*=.111
Diuretics [*N* (%)]	12 (8%)	8 (17%)	$\chi^2(1)=2.788$, φ=.120	*p*=.095
L-thyroxine [*N* (%)]	9 (6%)	5 (10%)	$\chi^2(1)=.976$, φ=.071	*p*=.323
Lipid-lowering drugs [*N* (%)]	7 (5%)	6 (13%)	$\chi^2(1)=3.431$, φ=.133	*p*=.064
Pulmonary hypertension therapy [*N* (%)]	2 (1.4%)	4 (8.3%)	$\chi^2(1)=5.844$, φ=.174	*p*=.016
Specific antiarrhythmics [*N* (%)]	4 (3%)	2 (4%)	$\chi^2(1)=.245$, φ=.036	*p*=.620
Digoxin [*N* (%)]	3 (2%)	2 (4%)	$\chi^2(1)=.642$, φ=.058	*p*=.423
Calcium antagonists [*N* (%)]	2 (1%)	2 (4%)	$\chi^2(1)=1.399$, φ=.085	*p*=.237
Entresto (*N* [%])	2 (1%)	2 (4%)	$\chi^2(1)=1.399$, φ=.085	*p*=.237
Oral corticosteroids [*N* (%)]	3 (1%)	1 (2%)	$\chi^2(1)=.000$, φ=.001	*p*=.990
Antidiabetics [*N* (%)]	3 (1%)	0 (0%)	$\chi^2(1)=1.002$, φ=-.072	*p*=.317
Other antihypertensive medication [*N* (%)]	2 (1%)	1 (2%)	$\chi^2(1)=.121$, φ=.025	*p*=.728
Insulin [*N* (%)]	2 (1%)	0 (%)	$\chi^2(1)=.664$, φ=-.059	*p*=.415
Immunosuppressants [*N* (%)]	1 (1%)	0 (%)	$\chi^2(1)=.330$, φ=-.041	*p*=.565

Chi-Square Tests were performed and asymptotic two-tailed *p*-values are shown. $p \leq .05$ was considered statistically significant.ACE, angiotensin converting enzyme; AT, angiotensin; CV, cardiovascular.

Supplementary Results - Sensitivity Analysis

The following results are based on a sample that, compared to analyses presented in the main part of the manuscript, included all patients with a HADS-D score < 6. Therefore, patients with no symptoms of depression as well as patients with mild symptoms of depression based on previously reported cut-off values for ACHD patients, were included (1). The following analyses include data from $N = 327$ individuals. Group comparisons based on sleep duration are summarized in Supplementary Table 2.

Supplementary Table 4: Group comparisons of sociodemographic-, CV-, and psychological parameters based on sleep duration.

	**Sleep duration**			
	**> 6 h (*N*=235)**	**≤ 6 h (*N*=92)**	**Statistics**	***p*-value**
Age (years)	34.0 [27-40]	39.5 [33-49.5]	U=7490.5, Z=-4.321	p<.001
Female sex (*N* ([%])	116 (49%)	48 (52%)	$\chi^2(1)$=.209, φ=-.025	p=.647
BMI (kg/m^2)	24.4 [21.8-27.3]	25.1 [22.6-28.5]	U=9569.5, Z=-1.614	p=.107
Bethesda scale			$\chi^2(2)$=.063, φ=.014	p=.969
Bethesda I (*N* ([%])	21 (9%)	9 (10%)		
Bethesda II (*N* ([%])	82 (35%)	32 (35%)		
Bethesda III (*N* ([%])	130 (56%)	50 (55%)		
NYHA classification			$\chi^2(3)$=6.808, φ=.144	p=.078
NYHA I (*N* ([%])	177 (75%)	57 (62%)		
NYHA II (*N* ([%])	43 (18%)	28 (30%)		
NYHA III (*N* ([%])	14 (6%)	7 (8%)		
NYHA IV (*N* ([%])	1 (0.4%)	0 (0%)		
Beta blockers (*N* [%])	44 (30%)	22 (46%)	$\chi^2(1)$=3.965, φ=.143	p=.046
PH therapy (*N* [%])	2 (1.4%)	4 (8.3%)	$\chi^2(1)$=5.844, φ=.174	p=.016
Sleep duration (h/night)	7.0 [7.0-8.0]	6.0 [5.0-6.0]	U=.000, Z=-14.243	p<.001
HADS Depression scale	2 [1.0-3.0]	2 [1.0-4.0]	U=9586.0, Z=-1.624	p=.104
HADS Anxiety scale	4 [2.0-7.0]	5 [3.0-7.0]	U=9250.0, Z=-1.487	p=.137
HADS sumscore	6 [4.0-9.0]	7.5 [4.8-10.3]	U=9052.5, Z=-1.750	p=.080

If not indicated otherwise median and interquartile range (IQR) is depicted and asymptotic two-tailed p-values are shown. $p \leq .05$ was considered statistically significant. BMI, body mass index; HADS, hospital anxiety and depression scale; NYHA class, New York Heart Association Functional classification; PH, pulmonary hypertension.

EAT thickness and NT-proBNP levels in ACHD patients based on sleep duration and age

As described for the respective analyses in the main part of the manuscript, the variable age was dichotomized by median split and subsequently, two-way multivariate ANCOVA with EAT and NT-proBNP as dependent variables, sleep duration and age group as independent variable and sex, BMI, and NYHA class as covariates was performed to assess a potential association of sleep duration on prognostic markers of CVD.

Similar to results derived from the sample described in the main part of the manuscript, respective analysis based on the redefined sample using a cut-off < 6 indicated a significant effect of sleep duration on the combined dependent variables EAT and NT-proBNP ($F(2, 319) = 3.152$, $p = .044$, Wilk's $\Lambda = .981$). Additionally, a significant difference between age groups on the combined variables was found ($F(2, 319) = 7.901$, $p < .001$, Wilk's $\Lambda = .953$) and a significant interaction effect of sleep duration and age group was present ($F(2, 319) = 3.159$, $p = .044$, Wilk's $\Lambda = .981$).

Post-hoc univariate ANCOVAs failed to show a significant effect of sleep duration on EAT ($F(1, 320) = 3.531$, $p = .061$, $\eta^2 = .011$) However, a significant effect on NT-proBNP ($F(1, 320) = 4.102$, $p = .044$, $\eta^2 = .013$) was found. Further, results indicate a significant difference between age groups for EAT ($F(1, 320) = 13.446$, $p < .001$, $\eta^2 = .040$), as well as for NT-proBNP ($F(1, 320) = 5.195$, $p = .023$, $\eta^2 = .016$). Similar to the results described in the main part of the manuscript, a significant interaction effect for sleep duration and age group was shown for NT-proBNP ($F(1, 320) = 5.975$, $p = .015$, $\eta^2 = .018$), but not for EAT $F(1, 320) = 1.204$, $p = .273$, $\eta^2 < .004$).

Pairwise group comparisons based on estimated marginal means and using with Bonferroni-corrected post-hoc test confirm results detailed in the main part of the manuscript and are depicted in Supplementary Table 3.

Supplementary Table 5: Pairwise group comparisons for EAT and NT-proBNP depending on age group and sleep duration

<table>
<tr><th>Dependent variable</th><th>Age group</th><th>Sleep duration</th><th>Mean ± SEM</th><th>Statistic</th></tr>
<tr><td rowspan="4">EAT (cm)</td><td rowspan="2">< 35 years</td><td>> 6 h</td><td>.342 ± .012</td><td rowspan="2">$p = .613$, $M_{Diff} = .013$, 95%-CI [-.036, .062]</td></tr>
<tr><td>≤ 6 h</td><td>.354 ± .022</td></tr>
<tr><td rowspan="2">≥ 35 years</td><td>> 6 h</td><td>.385 ± .012</td><td rowspan="2">$p = .020$, $M_{Diff} = .048$, 95%-CI [.008, .088]</td></tr>
<tr><td>≤ 6 h</td><td>.433 ± .017</td></tr>
<tr><td rowspan="4">NT-proBNP (ng/l)</td><td rowspan="2">< 35 years</td><td>> 6 h</td><td>237.3 ± 35.1</td><td rowspan="2">$p = .788$, $M_{Diff} = -20.3$, 95%-CI [-168.2, 127.6]</td></tr>
<tr><td>≤ 6 h</td><td>217.0 ± 66.7</td></tr>
<tr><td rowspan="2">≥ 35 years</td><td>> 6 h</td><td>232.3 ± 36.2</td><td rowspan="2">$p < .001$, $M_{Diff} = 217.4$, 95%-CI [95.9, 338.8]</td></tr>
<tr><td>≤ 6 h</td><td>450.0 ± 50.7</td></tr>
</table>

Estimated marginal means and 95% confidence intervals (calculated with BMI = 25.385, NYHA class = 1.35, and sex = 0.50) are depicted. Bonferroni-corrected two-tailed *p*-values for respective group comparisons are depicted. $p \leq .05$ was considered statistically significant.

References

1. Westhoff-Bleck M, Winter L, Aguirre Davila L, Herrmann-Lingen C, Treptau J, Bauersachs J, et al. Diagnostic Evaluation of the Hospital Depression Scale (Hads) and the Beck Depression Inventory Ii (Bdi-Ii) in Adults with Congenital Heart Disease Using a Structured Clinical Interview: Impact of Depression Severity. *Eur J Prev Cardiol* (2020) 27(4):381-90. Epub 20190726. doi: 10.1177/2047487319865055.
